# Early Evidence for the Extensive Heat Treatment of Silcrete in the Howiesons Poort at Klipdrift Shelter (Layer PBD, 65 ka), South Africa

**DOI:** 10.1371/journal.pone.0163874

**Published:** 2016-10-19

**Authors:** Anne Delagnes, Patrick Schmidt, Katja Douze, Sarah Wurz, Ludovic Bellot-Gurlet, Nicholas J. Conard, Klaus G. Nickel, Karen L. van Niekerk, Christopher S. Henshilwood

**Affiliations:** 1 PACEA, CNRS—University of Bordeaux, Pessac, France; 2 School of Geography, Archaeology and Environmental Studies and Evolutionary Studies Institute, University of the Witwatersrand, Johannesburg, South Africa; 3 Department of Prehistory and Quaternary Ecology, Eberhard Karls University of Tübingen, Tübingen, Germany; 4 Department of Archaeology, History, Cultural Studies and Religion, University of Bergen, Bergen, Norway; 5 MONARIS, Sorbonne Universités, UPMC Université Paris 6, UMR 8233, Paris, France; 6 Department of Geosciences, Applied Mineralogy, Eberhard Karls University of Tübingen, Tübingen, Germany; Universidade do Algarve, PORTUGAL

## Abstract

Heating stone to enhance its flaking qualities is among the multiple innovative adaptations introduced by early modern human groups in southern Africa, in particular during the Middle Stone Age Still Bay and Howiesons Poort traditions. Comparatively little is known about the role and impact of this technology on early modern human behaviors and cultural expressions, due, in part, to the lack of comprehensive studies of archaeological assemblages documenting the heat treatment of stone. We address this issue through an analysis of the procedure used for heating and a technological analysis of a lithic assemblage recovered from one Howiesons Poort assemblage at Klipdrift Shelter (southern Cape, South Africa). The resulting data show extensive silcrete heat treatment, which adds a new dimension to our understanding of fire-related behaviors during the Howiesons Poort, highlighting the important role played by a heat treatment stage in the production of silcrete blades. These results are made possible by our new analytical procedure that relies on the analysis of all silcrete artifacts. It provides direct evidence of a controlled use of fire which took place during an early stage of core exploitation, thereby impacting on all subsequent stages of the lithic *chaîne opératoire*, which, to date, has no known equivalent in the Middle Stone Age or Middle Paleolithic record outside of southern Africa.

## Introduction

The intentional heat treatment of silica rocks constitutes a major technological milestone in prehistory since the earliest developments of stone tool-making. It provides the first evidence of a transformative technology, i.e. transforming the physicochemical properties of a material for technical purposes, and it marks the emergence of fire engineering as a response to a variety of needs that largely transcend hominin basic subsistence requirements. Heat treatment of stone has long been documented in the prehistoric record as an intentional technical process used to improve the working quality of silica rocks and to enhance the sharpness and straightness the tool edges [1–3]. This technological process was reinvented many times in the Upper Pleistocene and Early Holocene in various geographical contexts. Its first occurrence was recently pushed back to more than 70 ka (thousand years) ago in the South African Middle Stone Age (MSA) sequences from Pinnacle Point [4] and Blombos Cave [5]. The heat-treated raw material was silcrete, a continental silica rock [6] of rather good quality that acquires even better knapping quality when heated. The development of a fire-based transformative technology adds a new component to the extensive list of inventive solutions introduced in the MSA, in particular by Still Bay and Howiesons Poort groups [7, 8]. However, southern African MSA heat treatment of stone still remains poorly documented and much of the debate has focused on the heating methods and on the induced physical transformations of the silcrete [4, 9–12]. Conversely, very little is known about the role and impact of this new technology on early modern human behaviors and cultural expressions. In other words, the role of heat treatment in the MSA technological repertoire still has to be determined. The question remains whether this early emergence of stone heat treatment responds to a new set of specialized technological skills or whether it is part of the domestic sphere of activities. In this paper we address this issue through the analysis of the heating technique and technological strategy developed in a recently discovered and excavated MSA site: Klipdrift Shelter (KDS) (southern Cape region, South Africa) [13], a site that indicates the extensive use of fire for the heat treatment of silcrete within one discrete occupation layer of this site.

To date, evidence for heat treatment of stone has been described for a few South African MSA sites: Pinnacle Point, Blombos Cave, Diepkloof Rock Shelter and Mertenhof Shelter [4, 5, 9, 11, 13, 14]. They all are stratified sites with successive occupations devoted to a broad range of subsistence, technical and symbolic activities [4, 5, 13, 15–27]. Stone-heating is amongst the multiple technological and domestic uses of fire performed by the Still Bay and Howiesons Poort groups [28]. The widespread use of fire, including the repeated burning of living floors in some sites [29, 30], results in archaeological deposits that are extensively burnt. Thus, assessing the role played by fire with more precision is complicated by the fact that a large number of archaeological items in these sites were incidentally burnt after use. In this context, a crucial issue is to differentiate the post-discard burning events, resulting in what we call burnt artifacts, from artifacts that were intentionally heat-treated, referred to as heated artifacts.

Silcrete is the only raw material for which chemical and structural modifications through heat treatment by MSA groups has been observed in South Africa. While silcrete's thermal transformations are comparable to the ones of flint, it has been shown experimentally that the control of temperature and heating speed is significantly less demanding for silcrete than for flint [11, 31]. The presence of tempering-residue on some heated artifacts from Diepkloof further indicates direct contact between embers and silcrete during the heat-treatment and the use of a fast-heating process [11]. These data provide an interesting alternative to the model of a complex heating procedure involving the use of a sand-bath heating environment [4, 10]. Silcrete is a highly diverse category of rock, comprising a variety of types that differ in terms of texture, homogeneity and color. The visual results of silcrete's heat-induced transformations vary significantly from one silcrete type to another, complicating the identification of heat treatment. This difficulty is probably the reason why most studies on heat-treatment are limited to small quantities of so-called "diagnostic artifacts". A comprehensive understanding of the technological behaviors related to the heat treatment of silcrete, its role and impact within the lithic *chaîne opératoire*, is thus still missing.

In this paper we present a novel approach applied to all silcrete artifacts (>2 cm) from layer PDB at KDS. Our study is based on a piece-by-piece comparison, a non-destructive analysis that allows for the identification of all intentionally heated lithics. The methodological protocol used to identify the heated pieces is combined with the characterization of the technological stages or operations related to the heating process. This analysis provides new insights into the technological use of fire by MSA groups.

## Materials and Methods

### Archeological context

**Fig 1:**
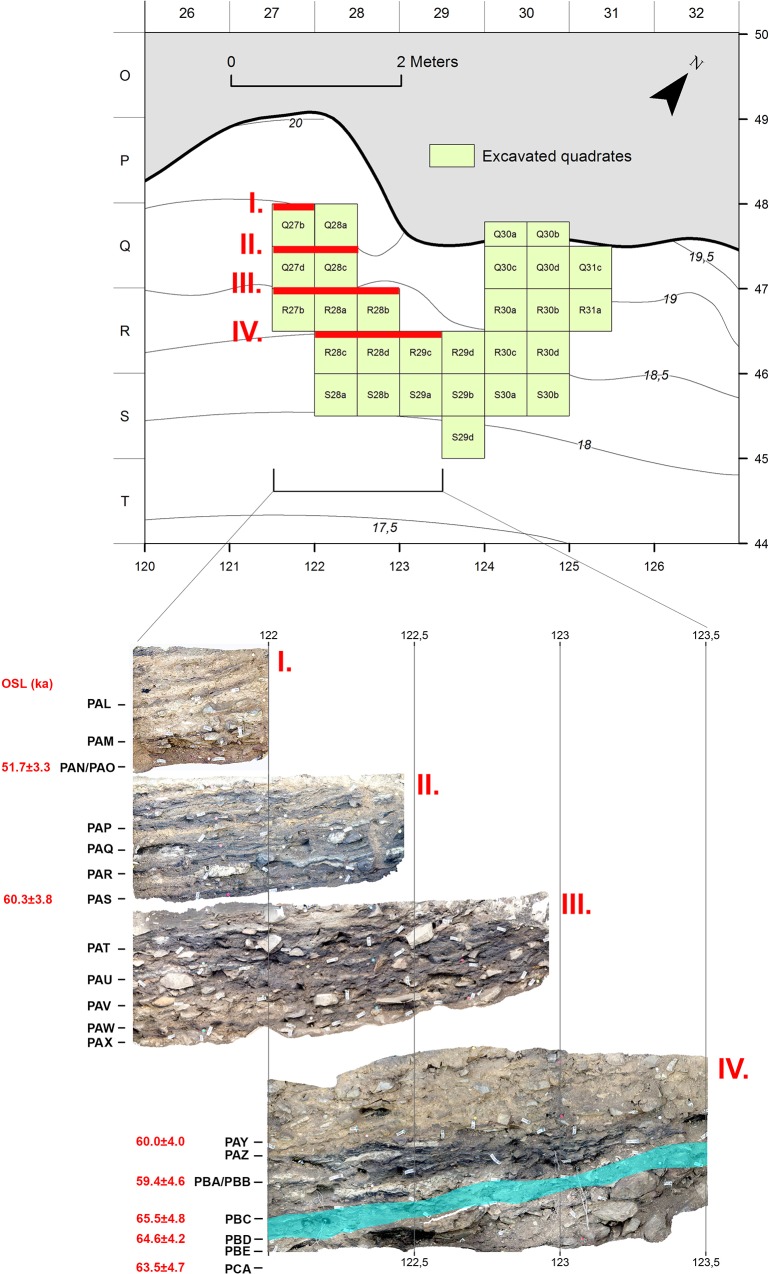
Map of the excavated area at KDS and site stratigraphy with OSL dates (illustration credit: Magnus M. Haaland, university of Bergen).

Silcrete forms a significant component of the raw materials used for tool production in some of the Howiesons Poort and Still Bay sites located on the western and southern Cape coasts of South Africa. The reason for this is the proximity of this zone to the so-called silcrete Cape coastal belt formed by the Cape Fold Mountains, where primary sources are relatively abundant [32]. Fine-grained silcrete outcrops occur in several localities at a minimum distance of 10 km north of KDS. This archeological site is part of the Klipdrift Complex, a series of sites located along the Indian Ocean shoreline in the De Hoop Nature Reserve, southern Cape region (Fig 1). KDS has been excavated since 2011 by C.S. Henshilwood and K.L. van Niekerk [13] with a permit obtained from Heritage Western Cape, the Provincial Heritage Agency based in Cape Town, South Africa. The research permits to conduct archaeological excavations are issued under the National Heritage Resources Act (Act 25 of 1999) and the Western Cape Provincial Gazette 6061, Notice 298 of 2003. CSH is the permit holder for the relevant permit: HWC REF No. HM / OVERBERG / CAPE AGULHAS / DE HOOP NATURE RESERVEI / KLIPDRIFT CAVE Permit No. 2010/06/001. No ethics clearance or permit is required to study the lithic artifacts from the Klipdrift Complex. The specimens used in this article are in a public collection that is accessible to other researchers. The lithics are housed and curated by the Iziko Museums of South Africa in Cape Town, as well as at Wits satellite laboratory in Cape Town. The lithics are catalogued under the labels: KDS 2011, 2012 and an individual record number is attributed to each artifact. A permit for transport and destructive analysis of 12 KDS artifacts studied here (Table 1) was provided by the South African Heritage Resources Agency (SAHRA) (PERMIT NO: 9/2/079/0008). All destructive and spectroscopic analyses reported in this paper fall under this permit. All necessary permits were obtained for the described study, which complied with all relevant regulations.

**Table 1:** Sample numbers and descriptions of the artifacts analyzed destructively and by IR spectroscopy.

Accession number	Observed heat treatment proxies	Performed analysis
KB421	Pre-heating scar, post-heating scar, tempering-residue	IR-ATR, Residue section
K2782	Post-heating scar, tempering-residue on natural surface	Thin section, IR-ATR
KB565	Pre-heating scar, post-heating scar, HINC	Thin section
K2840	Pre-heating scar, post-heating scar, tempering-residue	Thin section, IR-ATR
KB576	Pre-heating scar, post-heating scar, HINC	Thin section
K1938	Pre-heating scar, post-heating scar	Thin section
KB1817	Pre-heating scar, post-heating scar	Thin section
K2714	Pre-heating scar, post-heating scar	Thin section
KDS-1 (unpl.)	Pre-heating scar, post-heating scar	Thin section
KDS-2 (unpl.)	Pre-heating scar, post-heating scar, HINC	Thin section
KDS-3 (unpl.)	Pre-heating scar, post-heating scar	Thin section
KDS-4 (unpl.)	Pre-heating scar, post-heating scar	Thin section
KDS-5 (unpl.)	Post-heating scar, tempering-residue on natural surface	IR-ATR

unpl. = not piece-plotted during excavation. HINC = Heat-induced non-conchoidal fracture

The c. 1.2 m deep sequence is dated to between c. 70 and 50 ka by single-grain Optically Stimulated Luminescence (OSL) [13]. All layers contain well preserved archaeological assemblages, including marine and terrestrial faunal remains, organic materials, lithics, ochre and engraved ostrich eggshells [13]. Layers PCA to PAY relate to the Howiesons Poort industry. Silcrete is the main raw material used for blade production in the lower part of the sequence (layers PCA, PBE, PBD). The silcrete is predominantly fine-grained. The other materials with good knapping quality are fine-grained silica rocks (henceforth called chert) and calcrete. Both are poorly represented whereas local hydrothermal quartz and quartzite together account for more than half of the raw material used for knapping [13]. Considering that most hydrothermal quartz and quartzite cannot be expected to show any significant modification of their internal structure and knapping quality after heating, silcrete is the only good candidate for assessing the significance and role of heat treatment at KDS. Layer PBD, dated to 64.6±4.2 ka, was selected for this analysis because it contains the richest lithic assemblage (c. 2.500 artifacts > 2 cm) excavated from a total surface of 4 m^2^ including abundant silcrete artifacts. In layer PBD silcrete accounts for 46% of the knapped component, the remaining raw material spectrum being composed of quartzite (35.4%), hydrothermal quartz (17.1%), chert and calcrete (1.5%). Blade production is the unique target of the silcrete reduction sequence (n = 531/862, 61.6% of blades), while also being well developed on quartz (n = 150/316, 47,5%) but being quite marginal on quartzite (n = 81/653, 12.4%) (for more details, see [13]). The assemblage includes end- and by-products from all stages of the *chaîne opératoire*, resulting from multiple on-site technological activities ranging from blank production to tool use and discard.

### Quantifying the prevalence of heat treatment of silcrete

When silcrete is heated, it undergoes several chemical and physical changes. These changes include increased brittleness [33], occasional heat fracturing [34], reddening [35] and the loss of porosity [11]. Thus, past heating of stone may be identified through archeometric techniques. However, identifying these characteristics does not directly imply intentional heat treatment because unintentional burning and taphonomic processes such as natural fires at the site cause identical transformations in silcrete. Therefore, recognizing lithic heat treatment must follow a line of reasoning that aims to show intentionality, for example, by demonstrating that rocks were systematically and repeatedly knapped after their heat-treatment. In order to investigate post-heat treatment knapping, the signature of a fracture that took place after heat treatment (a post-heating scar) has to be recognized and distinguished from a fracture that occurred in unheated material (a pre-heating scar). In silcrete, this distinction is generally possible because heat treatment causes structural transformations that allow the subsequent fractures to propagate more evenly [11]. The resulting fracture surfaces show less micro- relief (i.e. are smoother) than fracture surfaces on unheated silcrete. Hence, in principle, post-heating removal scars can be distinguished from pre-heating scars by their relatively increased smoothness. The approach to measure “gloss” [4] on silcrete artifacts for determining whether these artifacts were heat-treated or not is based on the same principle. Gloss on knapped fracture surfaces is controlled by several factors, one of which is the microscopic roughness of the surfaces that causes incident light to be more or less reflected back. The light reflection, or gloss, is therefore an indirect measure of the micro-topography of fracture surfaces. However, the reflection of light is also controlled by the refractive index and the absorption coefficient, two material properties put into relation by the Fresnel equations [36]. Mineralogical composition therefore influences the measured gloss values. For example, variations of the anatase (TiO_2_) concentration, common in South African silcrete [6, 32], influence the total light reflection from the silcrete surface because of the relatively high refractive index of anatase. This can be expected to result in different gloss values from two silcrete types, even though they have similar surface roughness values. This is why we prefer to directly observe the surface roughness and use the terms ‘smooth’, as the opposite of ‘rough’ instead of ‘glossy’ and ‘dull’.

**Fig 2:**
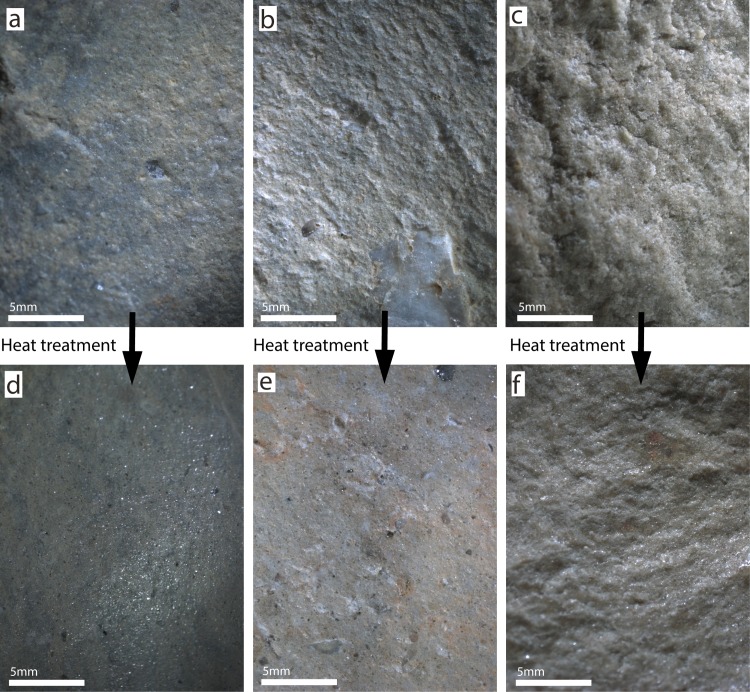
Comparison between fracture surfaces from before and after heat treatment. (a, b, c) = before heat treatment, (d, e, f) = after heat treatment. SK-13-03C before (a) and after (d) heat treatment; SK-13-04C before (b) and after (e) heat treatment; SK-13-03B before (c) and after (f) heat treatment. Note that, regardless of the initial roughness of the silcrete type, heat treatment results in smoother fracture patterns but pre-heating fracture scars on fine-grained silcrete (a) may be smoother than post-heating scars on coarser silcrete types (f). All fracture surfaces were photographed at identical magnification and in similar raking light conditions.

The apparent advantage of gloss analysis over the visual estimation of surface roughness is that it produces absolute values that can be used to demonstrate whether an assemblage is likely to contain heat-treated artifacts or not. However, single artifacts cannot be identified as being heat-treated or not heated with this method. The reason for this is the structural heterogeneity of South African silcrete types. The silcrete types used for stone knapping in the MSA range from very fine matrix-supported rocks with almost no inclusions to very coarse grain-supported types with up to 4% TiO_2_, resulting in very different natural fracture patterns and gloss values, even without heat treatment. Without the knowledge of the exact silcrete type, it is therefore not possible to interpret the gloss value obtained on a single artifact. The same is true for a visual estimation of surface roughness without prior distinction of different silcrete types. Relatively rough silcrete types, even when heat-treated to high temperatures, may display post-heating scars that are rougher than pre-heating scars of finer silcrete types. The comparison of Fig 2A with Fig 2F illustrates this problem by showing the contrast between a removal scar on a fine, but not heated piece of silcrete (Fig 2A) and a post-heating scar on another, coarser but heat-treated piece of silcrete (Fig 2F). Here, the direct measurement of gloss or the estimation of surface roughness, without the comparison with a meaningful reference collection that contains the different silcrete types would have yielded an erroneous result.

Such problems can be overcome if pre- and post-heating scars are preserved on a single artifact: the most reliable way to identify post-heating scars is to directly compare their smoothness with relatively rougher pre-heating scars on the same artifact. However, such pieces are not the majority in heat-treated assemblages, where a large number of artifacts show post-heating scars only [9] and consequently no contrast in roughness. In these cases, the artifacts must be compared to a geological reference sample of silcrete (unheated and experimentally heat-treated) with the same structure and micro-facies, hence with the same fracture properties as the artefact, in order to determine whether the observed flake scars are pre- or post-heating scars. When choosing such reference samples, it is important to take into account the characteristics that drive the different fracture patterns in silcrete: texture type, clast grain-size, crystallography and grain-size of the matrix, inclusions and alterations of the texture, etc. This is the approach that we apply to the study of the KDS silcrete artifacts.

### Archeological reference samples

12 silcrete artifacts that represent the macroscopic variability of silcrete types in layer PBD were selected for destructive analysis because of the presence of distinct markers of intentional heat treatment. Petrographic thin sections were cut from 11 of them. Accession numbers and brief descriptions of the archaeological samples analyzed destructively and by infrared (IR) spectroscopy are summarized in Table 1.

### Geological reference samples

**Fig 3:**
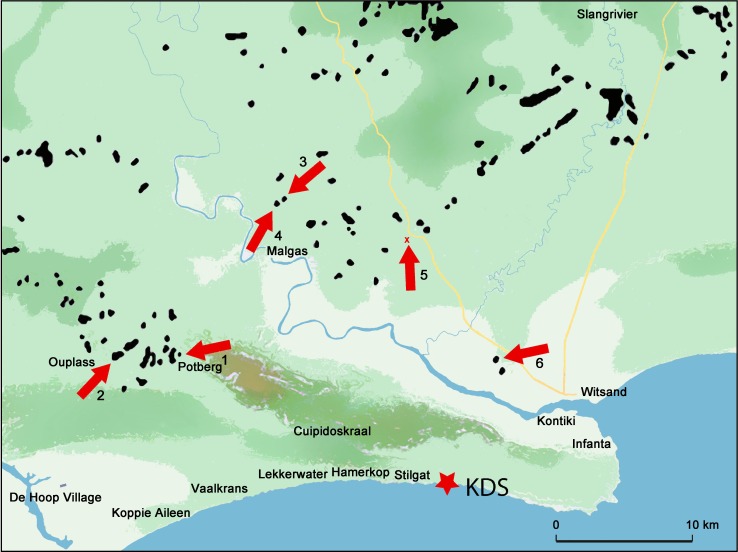
Map of the KDS area showing the six sampling locations for our reference collection (illustration credit: Gauthier Devilder, PACEA/CNRS-university of Bordeaux). The black spots correspond to primary silcrete and ferricrete outcrops georeferenced by Roberts (2003). The red x of source 5 indicates that this outcrop was not georeferenced by Roberts.

In order to identify and to assess the presence/absence of flake scars from before and after heat treatment on the PBD artifacts, we built a reference collection of unheated and experimentally heat-treated flakes made from comparable silcrete types (S1A and S1B Fig). For this, we sampled different silcrete types in the vicinity of KDS. We collected 31 samples from six different locations (Fig 3) and cut petrographic thin sections from each specimen in order to compare them with the thin sections of the archaeological samples shown in Table 1. The purpose of this sampling project was not to propose a raw material provisioning scheme for KDS but to collect geological reference material with a comparable structure, and hence, with comparable thermal behavior, for building the reference collection.

First, we compared the microfacies observed in the archaeological thin sections with the microfacies observed in the 31 geological thin sections. Criteria for this comparison were: texture type [6], clast grain size and sorting; degree of rounding, dissolution and overgrowth; cloudy iron oxide-, clay- or anatase-inclusions; presence/absence of indicators of Illuviation like colloform features [6] or clay skins. Only if all these criteria are identical in two distinct pieces of silcrete, do we expect that they contain similar amounts of molecular and chemically bound water [11] and hence, that they show similar thermal behavior. We removed one flake from each of these reference samples and then heat-treated the rest of the sample in an electrical furnace at 450°C for 3h with a ramp of 4°C/min. 450°C is near the upper limit of experimentally determined temperatures of heat treatment [9]. The atmosphere in the oven is expected to have no effect on the thermal transformations [11, 37, 38]. The ramp rate was chosen arbitrarily because it does not influence the thermal transformations as long as the sample is held at maximum temperature for > 1h [39], it only controls the probability of heat-induced fracturing [40]. A second set of flakes was removed from each sample after they had cooled to room-temperature. The flakes produced were used to recognize pre-heating and post-heating removal scars of the KDS PBD artifacts by directly comparing the latter with the corresponding silcrete types of the reference collection (as determined on the basis of macroscopic similarity).

**Fig 4:**
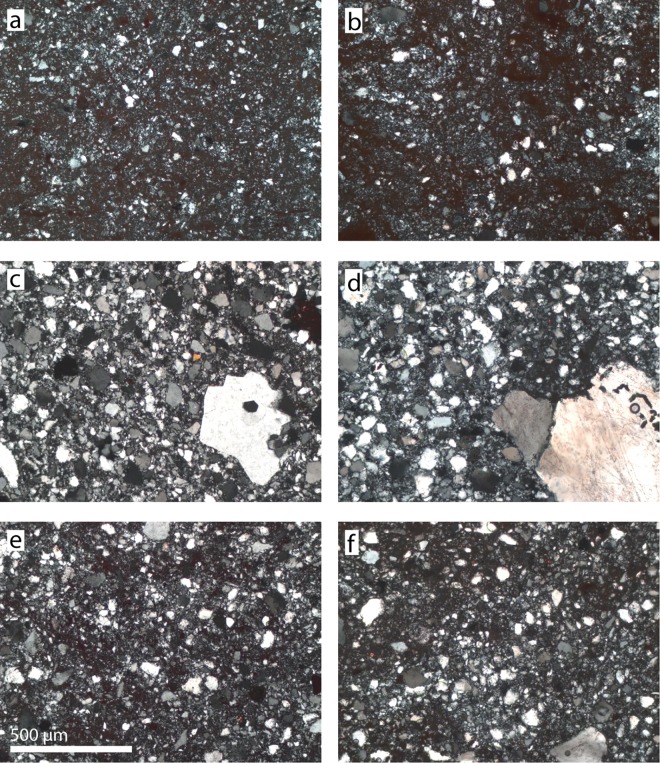
Micrographs of archaeological samples and geological samples with similar petrographic textures. (a, c, e) = Archaeological samples and (b, d, f) = geological samples. Cross polarized light. (a) K2782 and (b) its geological counterpart SK-13-01A, (c) K2714 and (d) its geological counterpart SK-13-03B, (e) K576 and (f) its geological counterpart SK-13-04C.

Fig 4 shows three examples of matching thin section micrographs used to identify meaningful reference samples. Because some of the geological samples showed almost identical microfacies, we selected 17 of them that correlated best to the 11 artifacts from PBD (Table 1). Control flakes were removed from these 17 reference blocks before they were heat-treated. Two of the blocks shattered during heating while the other 15 did not break and were perfectly suitable for knapping after the procedure. The fracture surfaces of the 15 flakes removed after heat treatment differ from the fracture surfaces of the unheated control flakes in that they are distinctively smoother. Fig 2 illustrates the transformation of the fracture pattern after heat treatment for three silcrete samples with different grain-size.

Thus, our experimental reference collection contains 30 flakes from 15 reference samples that are representative of the different silcrete types worked in PBD, i.e. one control flake with pre-heating scars and one heated flake with post-heating scars for each of the 15 samples. During the identification of heat treatment proxies on KDS lithics, these reference samples were laid-out on a table and each artifact was matched to one of the 30 flakes on macroscopic grounds. HINC fractures were identified on the basis of the criteria described in Schmidt et al. [9] and the two geological samples that shattered during heat treatment.

### Macroscopic identification of heat treatment proxies on artifacts

**Fig 5:**
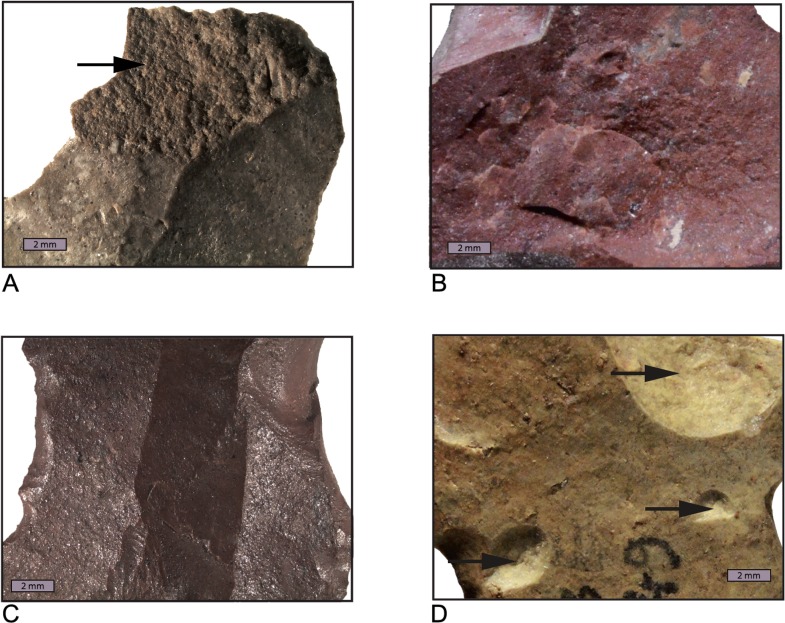
Macroscopic heating proxies. A: pre-heating scar distinguishable by the contrast between relatively rough and smooth surfaces, B: heat-induced non-conchoidal (HINC) fracture, characterized here by a non-conchoidal fracture plane with scalar features, C: post-heating scars, D: potlid fractures.

The identification of the heating proxies was performed for all silcrete pieces from layer PBD by direct comparison with the experimental reference collection. As proposed by Schmidt et al. [9], we identified the following proxies (Fig 5):

1. *Pre-heating scar* (Fig 5A): a relatively rough fracture surface corresponding to the removal of a flake from unheated silcrete.
2. *Post-heating scar* (Fig 5C): a relatively smooth fracture surface corresponding to the removal of a flake from heat-treated silcrete.
3. *Heat-induced-non-conchoidal (HINC) fracture* (Fig 5B): a fracture surface produced by failure during heating (sometimes also termed ‘overheating’ [40]). HINC fracture surfaces can be recognized due to their strong surface roughness, the presence of scalar features on the surface [9] and concave morphologies that often contain angular features. We only identify this type of fracture surface as HINC when it is clearly cross-cut by at least one post-heating removal. This technological relationship indicates that the failure occurred during heat treatment, i.e. that the reduction was continued afterwards. In the opposite case, when such a fracture surface is not cross-cut by a flake removal, it may result from fracturing at any stage, e.g. during accidental burning after discard, and no technological information concerning heat treatment can be retrieved from it. It is noteworthy that HINC fractures more likely occur during fast heating and that they are almost absent when silcrete is heat-treated with slow heating rates like in a sand-bath [9, 10, 40].
4. *Tempering-residue*: a black organic tar (wood tar) produced by dry distillation of plant exudations that was deposited on the silcrete surface during its contact with glowing embers during burning [9]. We only identify a black organic residue as tempering-residue if it covers a pre-heating removal scar or a natural surface and when it is cross-cut by a post-heating removal scar, otherwise, the residue may result from taphonomic factors after discard of the artifacts.

In addition to these proxies related to an intentional heating process, we also distinguish potlid fractures (Fig 5D) or micro-cracks which are formed after the discard of the lithics and are thus characteristic of incidental events of burning.

### Infrared spectroscopy and microstructure of the identified residues

In order to verify that the residue we observed on KDS PBD artifacts is indeed an organic tar, we conducted infrared analyses on four artifacts containing the residue. We used non-destructive micro-ATR infrared spectroscopy on their surfaces in order to test whether the black KDS residue is an organic compound (Table 1). For this purpose, we acquired spectra in the spectral region that contains the specific absorption bands of C-H bonds (C-H stretching bands) because such bonds are characteristic for organic compounds. A Bruker IRscope II microscope with a 20X germanium-ATR objective connected to an FT-IR Equinox 55 spectrometer was used for this infrared ATR surface analysis (maximum size of the analyzed area 100 μm^2^, spectra acquired between 2650 and 3150 cm^-1^, resolution 2 cm^-1^).

After determining the organic nature of the black residue, we analyzed its microstructure and reflectance properties to confirm that it is wood tar. Because tempering-residue, as described by Schmidt et al. [9], results from the distillation of plant exudations, it is deposited on the silcrete surface as a hot liquid. This mode of deposition results in degassing pores that may be preserved in the hardened black residue and the residue may include micrometre-sized charcoal inclusions due to the direct contact of the silcrete with glowing embers. The reflectivity of wood tar is also characteristic [41] and can be recognized in reflected light microscopy. For analyzing these features, we cut sample KB421 perpendicularly to its pre-heating surface covered with the black residue. The so obtained section was then embedded in resin and polished to obtain a surface suitable for reflected light microscopy. We made microscopic observations of the sections at magnifications ranging from 100x to 500x, using a Leitz petrographic microscope setup for reflection and oil immersion.

### Technological description of the archeological artifacts

Our analysis of the heat-treated silcrete artifacts from KDS is based on a piece-by-piece macroscopic identification of the heating proxies described above, in combination with technological description. The identification of heating proxies was combined with a descriptive analysis of each artifact based on technological and size attributes. The technological description allows establishing the chronology of all knapping stages, from core preparation and blank production to tool retouch or use, while determining the chronological position of the heating proxies in this reduction sequence. The method developed here presents three key advantages: 1) it is based on objective and easily observable criteria; 2) it can be applied to complete assemblages; 3) it ensures reliable identification, by combining different heat treatment-related proxies with the technological stage to which each lithic artifact corresponds. Our study thus provides a complete characterization of the heated lithics from layer PBD at KDS.

## Results

### Scope of the silcrete heat treatment

In layer PBD, 92% of the silcrete pieces (n = 793/862 > 2 cm) show clear evidence of heating, while the remaining 8% (see Table 2) are mostly (7.2%) non-diagnostic pieces and 0.8% are unheated. The non-diagnostic pieces comprise artifacts incidentally burnt after discard as well as pieces corresponding to silcrete types for which the thermal transformations are not macroscopically distinguishable because of the absence of the respective silcrete type in our reference collection (S1A and S1B Fig). We can thus deduce that silcrete in layer PBD was extensively, if not entirely, heat-treated, which strongly argues for the intentionality of the heating process. By contrast, evidence of incidental burning is very low, with only 4% of artifacts burnt after discard (n = 31/793 heated artifacts). The latter are characterized by heat-induced alterations in the form of micro-cracks or potlid fractures, i.e. circular non-conchoidal hollows formed after the production of the artifacts and/or rough non-conchoidal surfaces covering the entire blanks. The burnt artifacts likely correspond to artifacts abandoned close to or in fireplaces after use.

**Table 2:** Comparative frequencies of the heated *vs* unheated or non-diagnostic component and of the heating proxies for the basic technological type-products.

	All silcrete		Blades		Flakes		Cores		Chunks	
	N	%	N	%	N	%	N	%	N	%
Post-heating removals	531	67	377	71	134	52.7	7	25	13	26.5
Pre-+post-heating removals	198	25	116	21.8	64	25.2	10	35.7	8	16.3
Heat-induced fracture	55	6.9	16	3	18	7.1	7	25	14	28.6
Tempering Residue	9	1.1	4	0.8	4	1.6	0	0	1	2.1
**TOTAL heated**	**793**	**92**	**513**	**96.6**	**220**	**86.6**	**24**	**85.7**	**36**	**73.5**
**TOTAL unheated/non-diagnostic**	**69**	**8**	**18**	**3.4**	**34**	**13.4**	**4**	**14.3**	**13**	**26.5**
**TOTAL**	**862**	**100**	**531**	**100**	**254**	**100**	**28**	**100**	**49**	**100**

**Fig 6:**
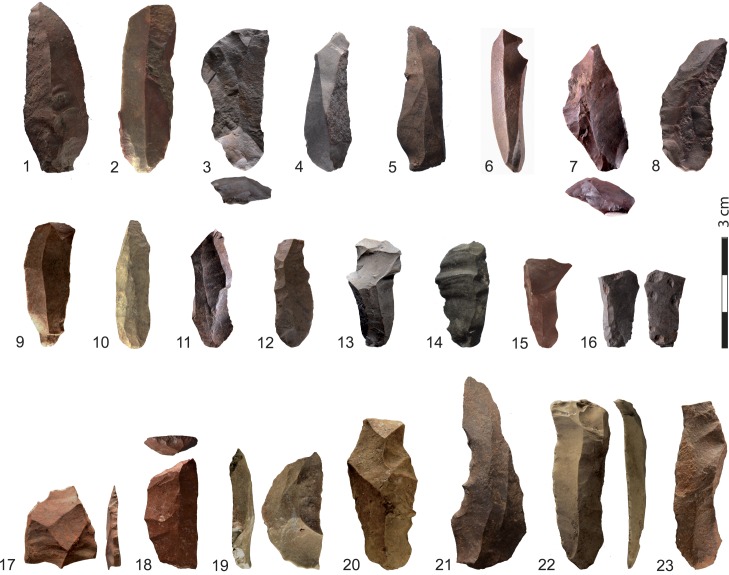
Heated blades and tools on blades from KDS, layer PBD. 1–16: silcrete blades showing their diversity in terms of size attributes and visual transformations after heating, 17–19: backed tools including one fragment of backed tool (17), one bi-truncated tool (18), one segment (19); 20,21: notched blades; 22,23: blades with slight continuous retouch on one lateral edge.

The heated component in layer PBD (Table 2) includes all categories of end-products and by-products. Blades (Fig 6) form the main category of end-product, and they are the exclusive target of the silcrete reduction sequence [13]. Their production conforms with what has already been described for other South African Howiesons Poort sites [42–46]. It is based on a marginal percussion technique using a soft hammer for the extraction of unidirectional blades from cores that are predominantly exploited on their larger flat surface. Blades are either unmodified and likely used as such, or more rarely transformed into a variety of formal tools (n = 24, 4.5% of all silcrete artifacts > 2 cm): backed tools and segments (n = 6, Fig 6: 17–19), blades with slight continuous retouch on one lateral edge (n = 8, Fig 6: 22, 23), notched blades (n = 2, Fig 6: 20, 21), borers (n = 2), *pièces esquillées* (n = 2) and miscellaneous tools (n = 4). A large majority of flakes are by-products of blade production (n = 218, 86%), resulting from core volume preparation, flaking surface and platform preparation, while only 14% (n = 36) are elongated flakes with blade negatives on their dorsal face and belong to a blade production stage. Chunks are waste products that result from knapping accidents either due to bad control of the force and/or location of the blow or to internal cracks in the raw material that have led to core fragmentation.

**Fig 7:**
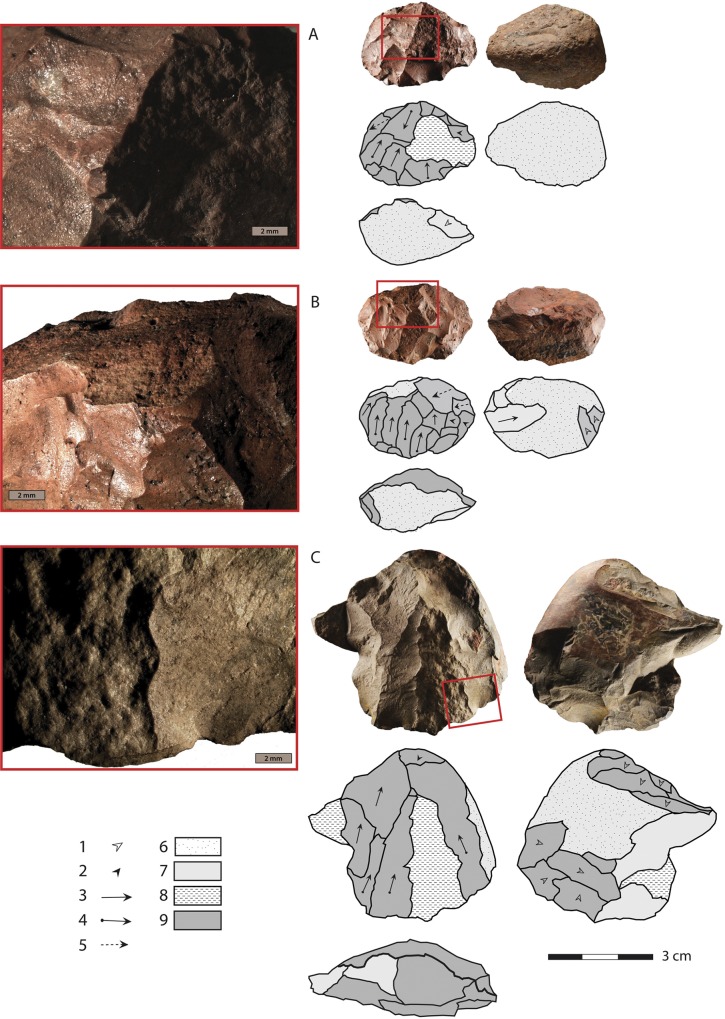
Heated blade cores from layer PBD, KDS. Caption for drawings: 1. knapping platform preparation, 2. convexity preparation, 3. blade removal without initiation, 4. blade removal with initiation, 5. indeterminate removal, 6. cortex, 7. pre-heating surface, 8. heat-induced non-conchoidal fracture (HINC), 9. post-heating removal. Note that all three cores (A, B, C) show a sequence of core exploitation (preparation and blade production) that follows a heat treatment which has resulted for A and C in heat-induced fractures, and which was preceded for A and B by a first stage of core exploitation.

**Fig 8:**
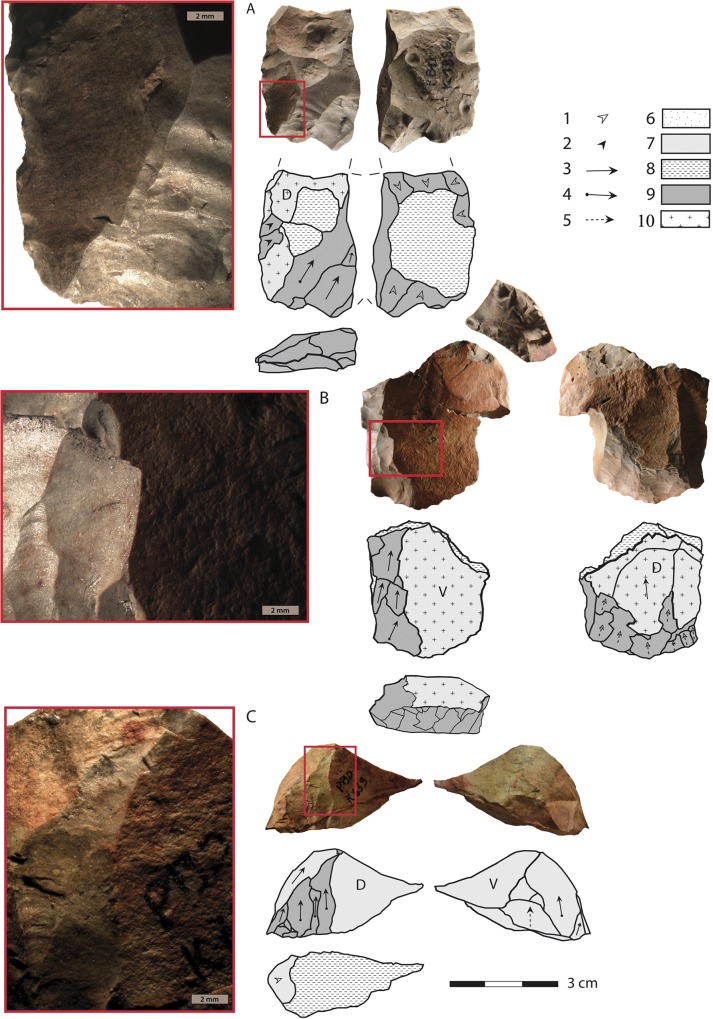
Heated blade cores from layer PBD, KDS. **Caption for drawings:** 1. knapping platform preparation, 2. convexity preparation, 3. blade removal without initiation, 4. blade removal with initiation, 5. indeterminate removal, 6. cortex, 7. pre-heating surface, 8. heat-induced non-conchoidal fracture (HINC), 9. post-heating removal, 10. patina. Note that all three cores present heat-induced non-conchoidal fractures; the heat-induced fracture visible on core B produced two refitted fragments, the biggest fragment was exploited as a core whereas the smallest was discarded. Core C shows a pre-heating knapping stage that includes core preparation and blade production. The initial blanks of cores B and C are flakes (V = ventral face, D = dorsal face).

The number of cores (n = 28), together with a core/blade ratio of 1/19 and a significant proportion of cortical elements (23.5%), suggest that the knapping sequence was performed on site, at least in part. A stage of core heating occurred during this sequence, as 24 of the 28 cores show evidence of heating before their reduction (Figs 7 and 8 and S2A–S2D Fig).

Among the diagnostic heating proxies observable on the artifacts, post-heating scars alone are by far the most frequent (531/793, 67% of all heated artifacts). The technological composition of this group is consistent with the overall composition of the heated artifacts (Table 2). It is indicative of long and extensive core reduction sequences that continue well beyond the first reduction stages which removed the surfaces initially heated. Post-heating scars thus encompass all stages of production, including core preparation (n = 69/531, 13%), blade production (n = 407/531, 76.5%) and retouch (26/531, 5%). The intensity of core reduction is also expressed by the fact that post-heating removals cover more than one half of the core flaking surfaces for a large majority of the cores (19/24 cores). These data are consistent with an extensive heating process that took place during an early stage of core exploitation and that impacted all subsequent stages, basically targeted at producing blades.

### Chronology of the heat treatment

Artifacts combining pre-heating and post-heating scars represent only 25% of all heated products, confirming the importance of not focusing solely on this category for assessing the role played by the heat treatment in an assemblage. They are nevertheless highly informative for assessing the chronological position of the heating stage relative to the whole lithic *chaîne opératoire*, as they provide information on the stages that directly precede and follow the heating stage (Table 2). The information here is more qualitative than quantitative as a significant proportion of the pre-heating surfaces are technologically undeterminable (n = 134/189, 71% of all artifacts with both pre-heating and post-heating scars, excluding the artifacts with pre-heating surfaces corresponding to cortex). This is due to the very small size of the surface areas or to the rough aspect of the pre-heating surfaces that prevent any accurate characterization of the corresponding scars. When readable, the pre-heating surfaces preserved on the products belong to a blade or flake production sequence (n = 41/189, 21.5%) or to a core preparation stage (n = 11, 6%), which means that some cores were flaked before heat treatment. The analysis of the cores confirms this observation. Some cores show pre-heating surfaces that correspond to blade or core preparation removals (Fig 7B and Fig 8C).Thus, in some cases the heating stage occurred after an initial stage of reduction, involving a segmented *chaîne opératoire* in which the heat treatment occurred during an early reduction stage, but not necessarily during the first stage of core exploitation.

### Technological advantages of heat-induced fracturing

Heat-induced fractures are quite frequent (7%) in layer PBD, and are observed on all categories of artifacts, including seven cores (Fig 7A and 7C and Fig 8A, 8B and 8C). A distinction has to be made between heat-induced fractures that occurred at an early stage of core exploitation and fractures that split cores or blanks into fragments at an advanced stage of transformation. While the latter may be interpreted as accidental and indicative of a total failure of the heating process, the former may not necessarily compromise the knapping process. In layer PBD, all artifacts with heat-induced fractures show negatives of removals which cross-cut the heat-induced fracture planes, which means that the flaking stages were subsequent to the heat-induced fracture of the core. Only a portion of the fragmented blocks actually preserves heat-induced fractures, and in some cases knapping must have removed them, which suggests that the real importance of heat-induced fractures is largely under-estimated in our accounts. A fairly large number of the raw material blocks must have therefore fractured during heat treatment, which strongly supports a procedure involving fast heating rates, although the exact quantitative pattern remains to be further experimentally tested.

The presence of silcrete cores that were exploited for blade production after an initial stage of heat-induced fracturing, combined with the total absence of cores abandoned right after a heat-induced fracturing, confirm that heat-induced fractures were not an obstacle for the KDS artisans. Heat-induced fractures also led to the production of large angular fragments suitable for initiating blade production with minimal core preparation. Portions of heat-induced fracture planes are visible on the flaking surface of the cores or on the platform preparation surface. The knappers took advantage of the fracture planes to rapidly start the blade production sequence, either by using the fracture plane as a platform for blade extraction without any further preparation (Fig 8C) or by directly exploiting the large surface created by the fracture for producing blades after a preliminary stage of platform preparation (Fig 7A and 7C). Fracturing of the block during heat treatment was thus a controlled risk, fully integrated in the core reduction sequence.

An additional advantage is that heat-induced fracture minimizes the risk of incidental fracture of the cores through internal heterogeneities or inclusions at a more advanced knapping stage. Iron oxide-hydroxide concentrations are common inclusions in the silcrete matrix [6], locally causing lower coherence between quartz grains that can induce knapping accidents. Such oxide-hydroxides produce H_2_O steam that may lead to preferentially fracturing the block at the zones where they are highly concentrated [9, 11]. The fragmentation may therefore be seen as resulting in a ‘cleaning’ of the blocks from unwanted inclusions. The controlled fragmentation of lithic raw material in a fire is a technical process also described in ethnographic records from North America [45, 46], the Andaman Islands [47] and Zimbabwe [48]. In the archaeological record, intentional heat-induced fragmentation of raw materials is also mentioned in the Sauveterrian culture of the French Mesolithic [49]. The intention of heat fracturing in these other contexts was to split the blocks for further knapping rather than to optimize the mechanical properties of the material.

**Fig 9:**
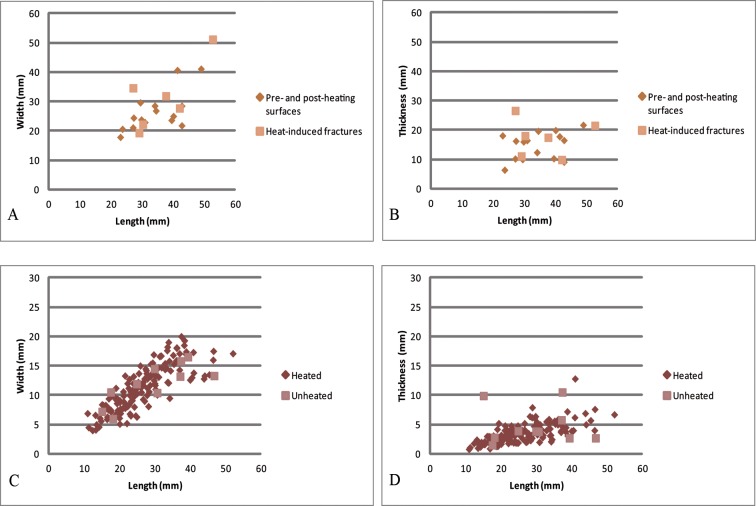
Size distribution of the cores and of the heated/unheated blades from KDS, layer PBD. A, B: length/width and length/thickness distribution of the cores showing heat-induced fractures compared with the cores showing only pre- and post-heating surfaces, C, D: length/width and length/thickness distribution of the heated blades compared with the unheated or non-diagnostic ones.

At KDS, it is impossible to assess whether the fragmentation of the silcrete blocks was intentional or whether the artisans opportunistically took advantage of the incidental fracturing of the blocks prior to their exploitation. The fact that knappers did not stop the knapping process after the core broke during heat-treatment suggests that it was, at least, an accepted risk. Heat-induced fragmentation of the blocks results in smaller proportions of chunks due to subsequent breakages and consequently higher blade productivity as observed when comparing the composition of the heated group with the unheated or non-diagnostic silcrete group (Table 2). It has been experimentally shown that larger volumes of heated raw material are associated with higher risks of fracture during the heating process [50, 51]. Core sizes are heterogeneous (Fig 9A and 9B), and range from very small sizes (maximum length < 30 mm) to small sizes (maximum length > 40 mm), with maximum length values around 50 mm. No direct correlation between size attributes and heat-induced fractures can be shown from the small sample of cores that show evidence for heat-induced fracturing (Fig 9A and 9B). As core dimensions only document the block volumes at their stage of discard, blade dimensions provide useful and complementary information (Fig 9C and 9D). Blade dimensions confirm the high heterogeneity of the lithic production in terms of size attributes. The blades range from very small, c. 12–13 mm maximal length, to large blades longer than 40 mm, with mean values of 26.4 mm (sd. 8.2), 11.3 mm (sd. 3.8) and 3.6 mm (sd.1.7) for length, width and thickness of the heated blades respectively. The size distribution of the unheated blades does not differ significantly (Fig 9C and 9D). Blades thus belong to a unique heterogeneous population, and no distinct bladelet group can be seen, although a significant proportion of blades (30%) are smaller than 20 mm in length. The diversity of blades is the result of a flexible system of blade production, as observed from the cores that include a variety of types: unifacial cores, multifacial cores, semi-rotating cores and cores exploited on their narrow face. We can deduce from this data that core heat treatment corresponds to the objectives of a flexible but nonetheless highly purposeful lithic production.

### Tempering residue

A small number of artifacts (1.1%), comprising blades, flakes and chunks, exhibit a black residue on pre-heating or natural surfaces that is visually similar to the tempering-residue described in Schmidt et al [9]. The residual deposit is located either on a portion of the outer natural surface of the initial blank or on a pre-heating surface. In either case, the residue-covered surfaces are cross-cut by post-heating removals. There is no evidence of residue on the post-heating surfaces. The residue was thus deposited on these surfaces after an initial stage of knapping and before the sequence of knapping that succeeded heat treatment, i.e. during the heating stage itself. If the nature of this black residue is confirmed to be tempering-residue, it indicates that silcrete blocks in KDS were heated in direct contact with embers in open-air domestic fires, as recently reported at Diepkloof Rock Shelter [9].

#### Infrared spectroscopy and reflection microscopy of the tempering-residue

**Fig 10:**
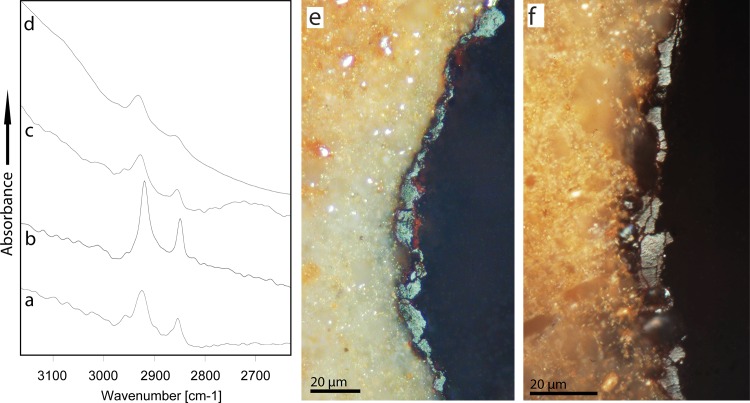
Micro-ATR infrared spectra and reflection photo-micrographs of KDS tempering-residue. Left: infrared spectra in the CH-stretching region: (a) K2782, (b) KB421, (c) K2840, (d) KDS-5. Spectra vertically offset for clarity. Right: reflection micrographs of a section of tempering residue on KB421. Note the low reflectance value of the residue (appearing as grey), indicating that it is formed by tar, and the pores close to the silcrete surface, indicate melt degassing during the formation of the tar as a hot liquid. Reflected white light and oil immersion.

All four infrared spectra of the black tempering-residue, covering pre-heating removal scars and natural surfaces on the four residue bearing artifacts, clearly show C-H stretching bands (Fig 10). The C-H band positions are not identical in all four samples but shift within a range of 10 cm^-1^, revealing structural differences in the CH bearing molecules. These differences may be due to various factors (temperature and duration of burning, differential taphonomic factors like oxidation, different types of plants used for fuel, etc.) and their interpretation lies beyond the scope of this work. However, the infrared spectra of the black deposit leave no doubt that the tempering residue observed on KDS artifacts is an organic compound.

In the polished KDS section, the residue appears to be a 1-to-20 μm-thick film on the silcrete surface (Fig 10). The low reflectance values of the material composing this film are close to the values known from wood tar [41]. Pores due to melt degassing are observable near the silcrete surface, indicating a deposition of the film on the silcrete surface as a hot liquid. Together with the organic nature, this microstructure supports the model of formations of tempering-residue as proposed by Schmidt et al. [9], i.e. the deposition of tar on the silcrete surface through dry distillation of the plant exudations (gum or resin) of the wood that is burned in the fire. Thus, our microscopic analysis of the residue on the KDS PBD artifacts supports that it is similar to the tempering residue on Diepkloof artifacts. However, no micro-charcoal inclusions can be observed in the tar as is the case for the Diepkloof samples. Whether this is due to differences in the plant species burned at KDS or due to variable locations of the silcrete within the hearths must await further microscopic analysis of supplementary artifacts with tempering residue.

## Discussion

### Methodological progress

Our approach of using a reference collection of silcrete with similar microfacies, and therefore similar thermal behavior, to identify heat treatment proxies on artifacts is a powerful non-destructive tool. Its application to the KDS PBD assemblage has proven successful as it allows the accurate characterization of all silcrete lithics in an assemblage. The results obtained by this technique are expected to be rather reliable because of the direct comparison between heated and not heated pieces of the same silcrete type. This approach is especially useful when the direct comparison between pre-heating and post-heating scars on a single artifact is not possible because only one of these types of removal scars is visible (i.e. the majority of artifacts). In view of the variability of silcrete types in South Africa, this approach can be expected to be more reliable than the use of a gloss meter [4].

However, 7.2% of all silcrete artifacts remained unidentified after our procedure. The reason for this is that these silcrete types did not macroscopically match one of the silcrete types in our reference collection, illustrating the need for exhaustive sampling of all silcrete types present in the studied archaeological assemblage. Another potential limitation of this approach would be long distance transport of silcrete [52]. Imported exogenous silcrete cannot be evaluated by our protocol because of the lack of appropriate reference samples.

### Technique used for heat treatment at KDS

During our comparison, we identified tempering-residue on some of the KDS artifacts. Structural and chemical analyses indicate that this residue has a similar origin as tempering-residue in Diepkloof [9]: an organic wood tar deposited on the silcrete surface as a hot liquid during heat treatment. The KDS PBD residue is associated with pre-heating removal scars or natural surfaces, which were both part of the outer surface of the silcrete blocks during their heat treatment. Post-heating-scars are free of black residue. Surfaces covered by the tempering residue are cross-cut by post-heating removal scars, indicating that the silcrete was knapped after the formation of the residue. This relation shows that the KDS tempering-residues do not result from taphonomic burning after discard. Although the overall frequency of tempering-residue appears low in layer PBD, it must be kept in mind that even if the total surface on a piece of silcrete is in contact with embers, only a small portion of this surface may be covered be tempering residue [9]. Thus, a relatively small percentage of artifacts showing traces of tempering residue may still be indicative of a large number of silcrete pieces heat-treated in embers. The model of ember-heat treatment is supported by the c. 7% of artifacts showing remnants of heat-induced fractures. As explained earlier, 7% of all heat-treated artifacts that show remnants of HINC fractures can be expected to indicate that a large number of the silcrete blocks brought to the site heat-fractured during heat treatment. Because such heat-induced fracturing during heat treatment is associated with fast heating rates like the ones produced in an open-air fire [9, 10] and because it is much rarer during slower sand-bath heating, the finding of HINC fractures on the PBD silcrete strongly suggests the use of a fast heating technique at KDS. It thus appears highly likely that heat treatment in the embers of open-air fires was a technique commonly used in layer PBD. Additionally, heat treatment in embers is currently the only archaeologically documented technique in the South African MSA [53] and at our given state of knowledge it also appears to be the best model for explaining heat treatment at KDS.

### Role and benefits of the heat treatment of silcrete at Klipdrift Shelter

The analysis performed on the lithics from layer PBD at KDS provides evidence of the extensive and structured use of fire as a transformative technology for silcrete knapping by some Howiesons Poort groups. It is a systematic procedure that results in more than 90% of the assemblage being intentionally heated. The heating stage occurred non-randomly, in an early stage of core exploitation, which was sometimes preceded by an initial knapping stage. As a consequence, the whole *chaîne opératoire*, from core preparation to blade production and tool manufacturing, benefited from the advantages of the heating process. The heat treatment of a lithic raw material from the earliest stages of lithic reduction, thereby impacting the entire lithic *chaîne opératoire*, is a practice which appears again much later in the prehistoric record, at the end of the Late Pleistocene [54].

The advantages of heat treating cores are multiple. As pointed out previously, heat treatment greatly facilitates silcrete knapping. The reason for this is similar to that for flint and chert whose heat treatment leads to a measurable reduction of the material’s fracture toughness [50] and to an increased hardness [51]. This means that less force is needed to detach a flake or blade after heat treatment, resulting in better control and precision during percussion. Improving the knapping quality of lithic raw material is a feature which seems common to all prehistoric cultures that intentionally heated silica rocks [3, 50, 55]. It was also very likely a major focus for some KDS artisans, as the extraction of blades using a soft hammer marginal percussion requires high striking precision. Additional advantages which seem specific to this assemblage relate to the heat-induced fracturing of the blocks at an early stage of core exploitation. These advantages include: 1) fragmentation, which results in the elimination of internal heterogeneities (iron oxide inclusions, remnants of illuviation features), which could have caused the incidental breakage of the core at an advanced stage of reduction; 2) the production of angular fragments with suitable angles and surfaces that can be directly exploited for knapping without further preparation; 3) fewer constraints on the selection of the volumes to be heat-treated. This heating procedure echoes the flexibility of the blade production system developed by the knappers. The flexibility is evident in the diversity of blade production methods and in the variability of blade size attributes. Our analysis of the lithics from layer PBD therefore shows that optimal control of the heating procedure, which would include avoiding breakage, was not a prerequisite for the KDS artisans (e.g.[10]).

Layer PBD relates to the intermediate Howiesons Poort phase as initially defined at Diepkloof Rock Shelter [22]. At KDS, this phase differs significantly from the late Howiesons Poort phase in tool types and raw material frequencies [13]. Silcrete is the predominant raw material and notched tools are the most common tools in the intermediate Howiesons Poort phase, while hydrothermal quartz is dominant in the late Howiesons Poort phase, in association with higher proportions of backed tools [13]. However, many elements of continuity exist from one layer to another at KDS. In particular blade production remains the primary objective of the lithic production in the whole Howiesons Poort sequence and silcrete heating appears as a constant feature, as observed from our preliminary analysis of the layers below (PCA, PBE) and above (PBC, PBA/PBB, PAZ, PAY) layer PBD. As the proportion of silcrete artifacts decreases in the upper part of the KDS sequence, it might be expected that heat treatment also becomes less prevalent.

### Heat treatment of silcrete during the MSA

The extensive heat treatment of silcrete in the Howiesons Poort is not only found at KDS. The recent analyses of the intermediate Howiesons Poort assemblages from Diepkloof Rock Shelter also indicate high proportions of heat-treated artifacts [9], over 90%, and thus similar to those observed at KDS in layer PBD. Evidence of heated cores and products in the Howiesons Poort layers of Pinnacle Point (PP5-6 site) suggests that silcrete heat treatment was also used at this site for blade production [4, 20, 56]. While detailed data are still required for in-depth comparative analyses of the heating practices and related technological behaviors, it seems clear that some Howiesons Poort groups who occupied these sites routinely heat-treated cores for producing blades. The KDS and Diepkloof records together suggest that the Intermediate Howiesons Poort phase, to which layer PBD belongs, would correspond to a period of extensive development of silcrete heat treatment for blade production. Considering that heating of lithic raw materials has only recently been described [4], further analyses are still required to test the presence of heating practices in a wider range of sites and time periods.

Silcrete heating by Still Bay artisans comprises a strategy that is different to that in the Howiesons Poort. At Blombos Cave, it was shown that the heating stage focused on tool manufacturing, in particular for the pressure retouch of the apical part of some Still Bay points [5]. Experiments have shown that silcrete pressure retouching cannot be performed without prior heat treatment of the raw material. Here, the benefit of heating is to allow and ensure the successful application of a specific technique that contributes to the final retouch of the apical part of points. Compared to the heating process in layer PBD at KDS, the heating process in the Still Bay at Blombos Cave is far more targeted. Although significantly different, the Still Bay and Howiesons Poort heating practices share the common feature of improving the knapping qualities of silcrete for the production of specific sought-after end-products, such as bifacial points and blades. Stone heating in the MSA is embedded in the on-site domestic activities that revolve around fire. It is worth noting that, at KDS, silcrete was collected from within a maximum radius of a few kilometers to a few tens of kilometers from the site whereas at Blombos Cave the silcrete sources were, according to our knowledge of present sources, located further away.

### Heating stone in prehistoric times: a recurrent and discontinuous practice

The Howiesons Poort heating practices, as defined in layer PBD at KDS, at Diepkloof Rock Shelter and Mertenhof Shelter, are unique and cannot be directly correlated with stone-heating practices documented in other prehistoric contexts. It is only from c. 20 ka that the heat treatment of stone developed in Asia and Europe [57]. Both in terms of heating method and technological achievement, the MSA heating practices differ significantly from the heating practices developed in the late Upper Pleistocene. In the Eurasian late Upper Pleistocene record, the emergence of flint heat treatment most likely implied setting up more complex heating structures, allowing a strict control of the heating temperature and rate [54, 57–61]. Beyond the recurrent association between heat treatment and industries targeting normalized end-products, such as blades, micro-blades or thin bifacial points, there is a significant technological gap between the MSA fire-based industries and the late Upper Pleistocene/Holocene fire-based cultures in Eurasia.

It has not been demonstrated so far that the MSA groups used complex heating methods. The presence of tempering residue, indicating heat treatment in direct contact with glowing embers, suggests that this procedure was often realized with a fast process using open fires. Another proxy strengthening the hypothesis of a fast heating technique is the fact that some of the artifacts show signs of fracturing during heat treatment (heat-induced fractures), because thermal fracturing more likely occurs during fast heating [10, 40]. The fire-based technology developed at KDS did not require highly specialized skills and was likely performed as part of on-site domestic activities. In the context of southern Africa, data are still too limited for exploring potential patterns of continuity or evolution across the MSA.

## Conclusion

Our data add a new dimension to the understanding of fire-related behaviors during the Howiesons Poort by demonstrating the major role played by heat treatment in the production of silcrete blades in layer PBD at KDS. It provides the first direct evidence of the intentional and extensive use of fire applied to a whole lithic *chaîne opératoire*, based on an analytical approach that has allowed the analysis of all heated lithics from layer PBD. The heat treatment of silcrete in this layer has impacted all stages of core reduction and all subsequent operations of tool manufacturing. For the artisans, the benefits of heat treatment performed in an early stage of the *chaîne opératoire* were multiple. The Howiesons Poort groups considerably developed and optimized a technology that had possibly emerged from the early MSA [4], and continued in the Still Bay (c. 77–72 ka) in relation to the Still Bay point manufacturing process[5].

Although further evidence is required, our analysis indicates that the heat treatment of silcrete was a major asset for some of the MSA groups who occupied the Cape region, by facilitating blade production in an area where the most abundant fine-grained raw material was difficult to exploit for such purposes. The heat treatment of silcrete thus reflects an innovative adaptation based on optimal use of local resources. The adaptable and innovative attitude was likely one factor that favored the widespread distribution of the Howiesons Poort material culture in southern Africa. Fire-based domestic activities were particularly well-developed in Howiesons Poort sites, and relate to various activities including site maintenance, hearth cleaning and burning of bedding [29, 30]. The regular occurrence of domestic hearths in the Howiesons Poort probably had a causal effect on the significant development of fire-based technological activities including heating for the preparation of compound-adhesives used for hafting tools [62–65] and making composite tools and stone-tipped arrows [66, 67]. Additionally, the heat treatment of silcrete, applied to the entire *chaîne opératoire* process, can be considered a significant innovation and was part of a package of new adaptive skills using fire that has no equivalent in the earlier Middle Stone Age or in the Middle Paleolithic.

## Supporting Information

S1 Fig Sample of silcrete reference collection used for comparison. A, B: illustration of experimental flakes struck before and after the heating of each block.
(PDF)

S2 Fig Silcrete cores from layer PBD. A, B, C, D: Picture and technological drawing of each core. Caption for drawings: 1. knapping platform preparation, 2. convexity preparation, 3. blade removal without initiation, 4. blade removal with initiation, 5. indeterminate removal, 6. cortex or patina, 7. pre-heating surface, 8. heat-induced fracture, 9. post-heating removal, 10. burnt after discard, potlids, 11. retouch.
(PDF)
