# Supplementary figures and images for: Early Evidence for the Extensive Heat Treatment of Silcrete in the Howiesons Poort at Klipdrift Shelter (Layer PBD, 65 ka), South Africa
Source: PLoS One. 2016 Oct 19;11(10):e0163874. doi: 10.1371/journal.pone.0163874 (PMC5070848; doi:10.1371/journal.pone.0163874)

A

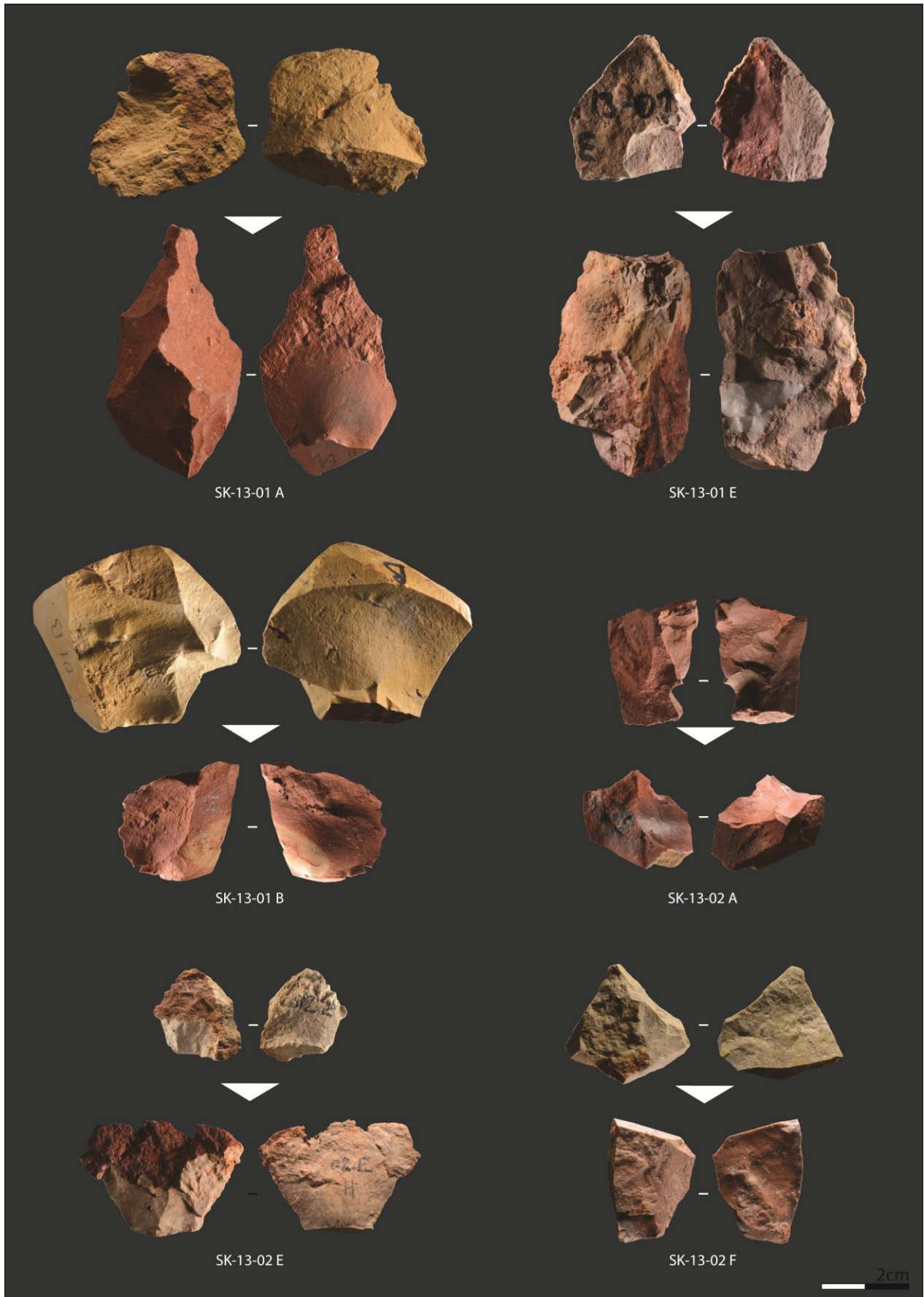

B

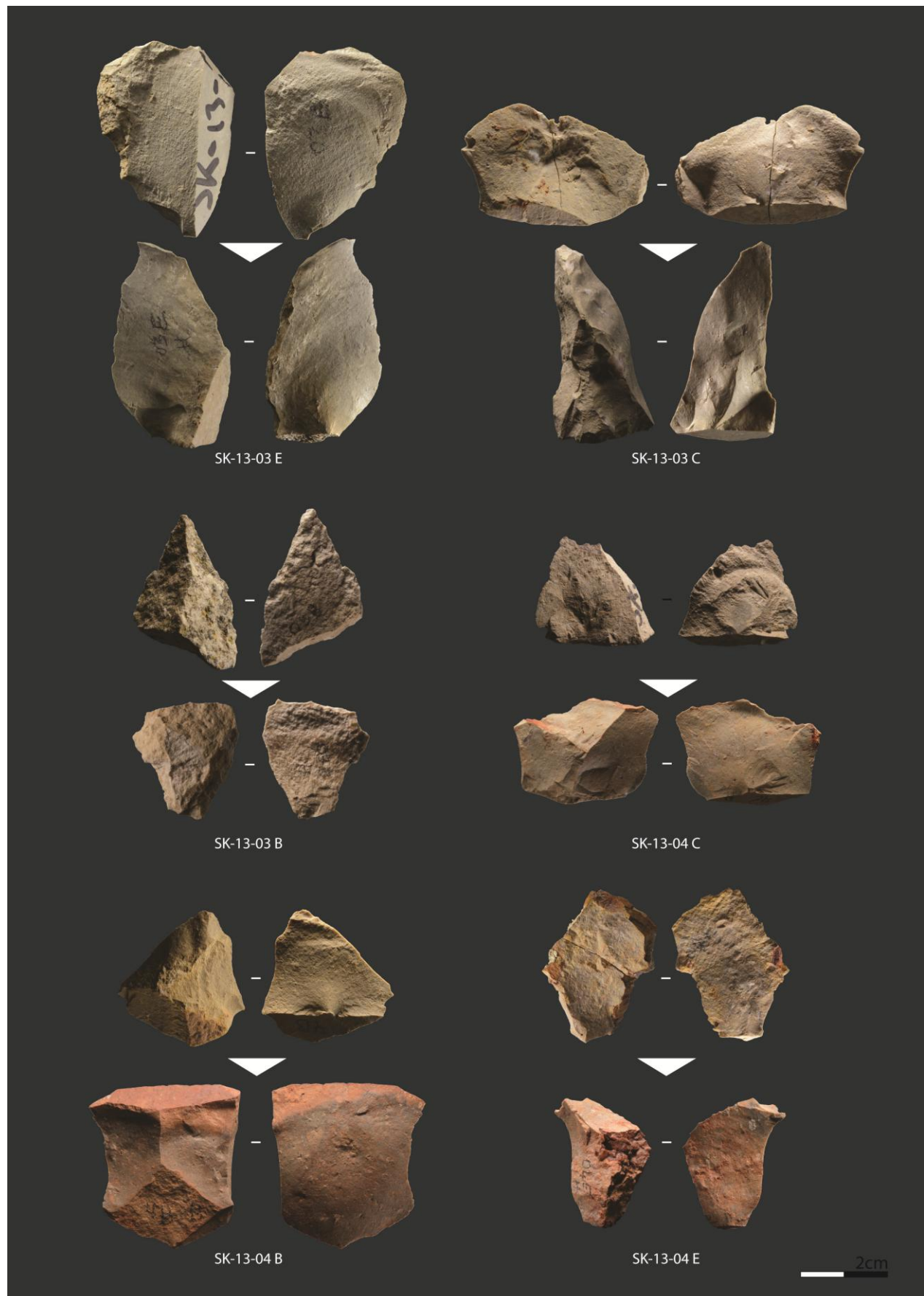

Supplement: S1 Fig — A, B: illustration of experimental flakes struck before and after the heating of each block. (PDF) [file pone.0163874.s001.pdf]
