# Supplementary material for: Early Evidence for the Extensive Heat Treatment of Silcrete in the Howiesons Poort at Klipdrift Shelter (Layer PBD, 65 ka), South Africa
Source: PLoS One. 2016 Oct 19;11(10):e0163874. doi: 10.1371/journal.pone.0163874 (PMC5070848; doi:10.1371/journal.pone.0163874)

A

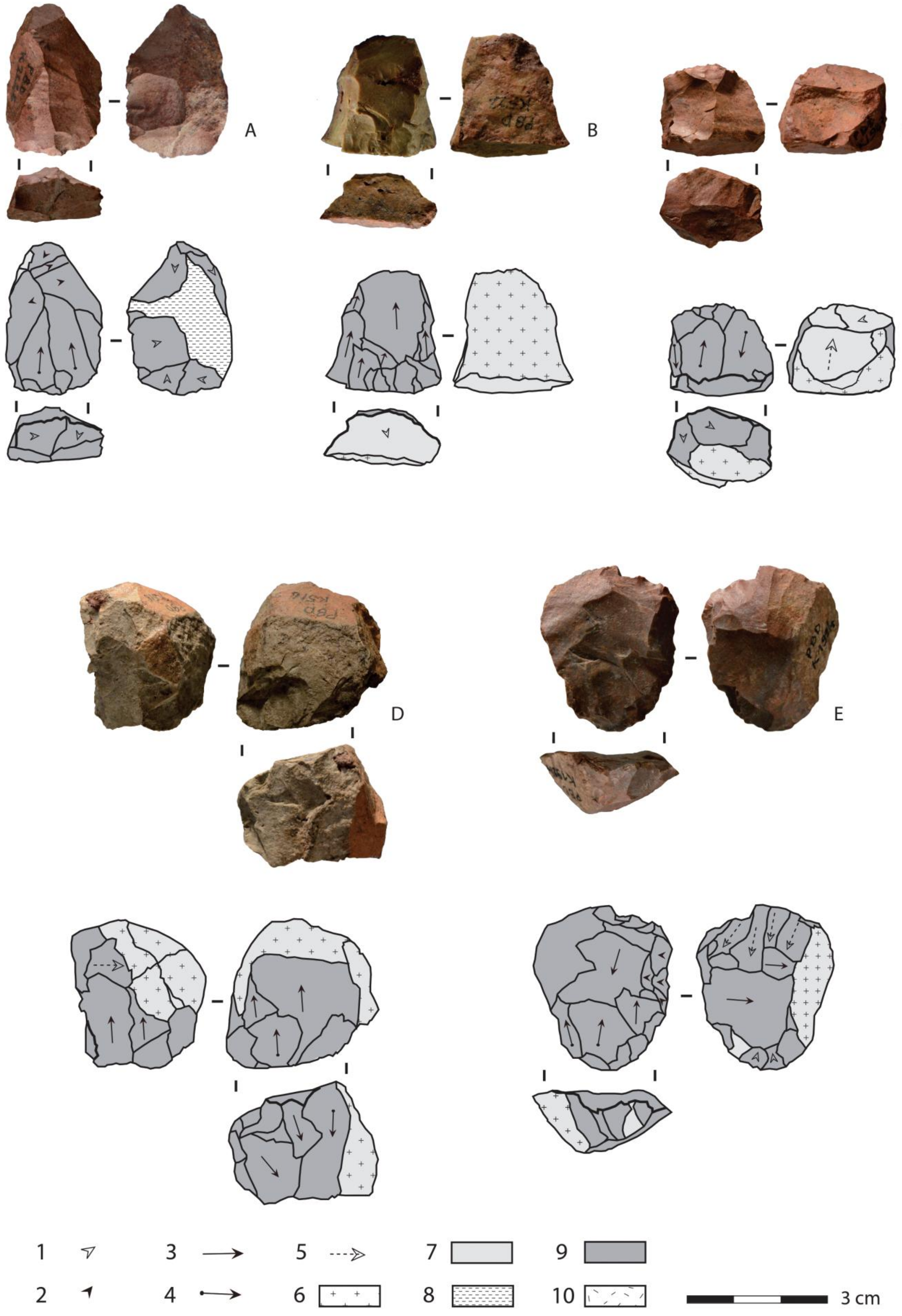

B

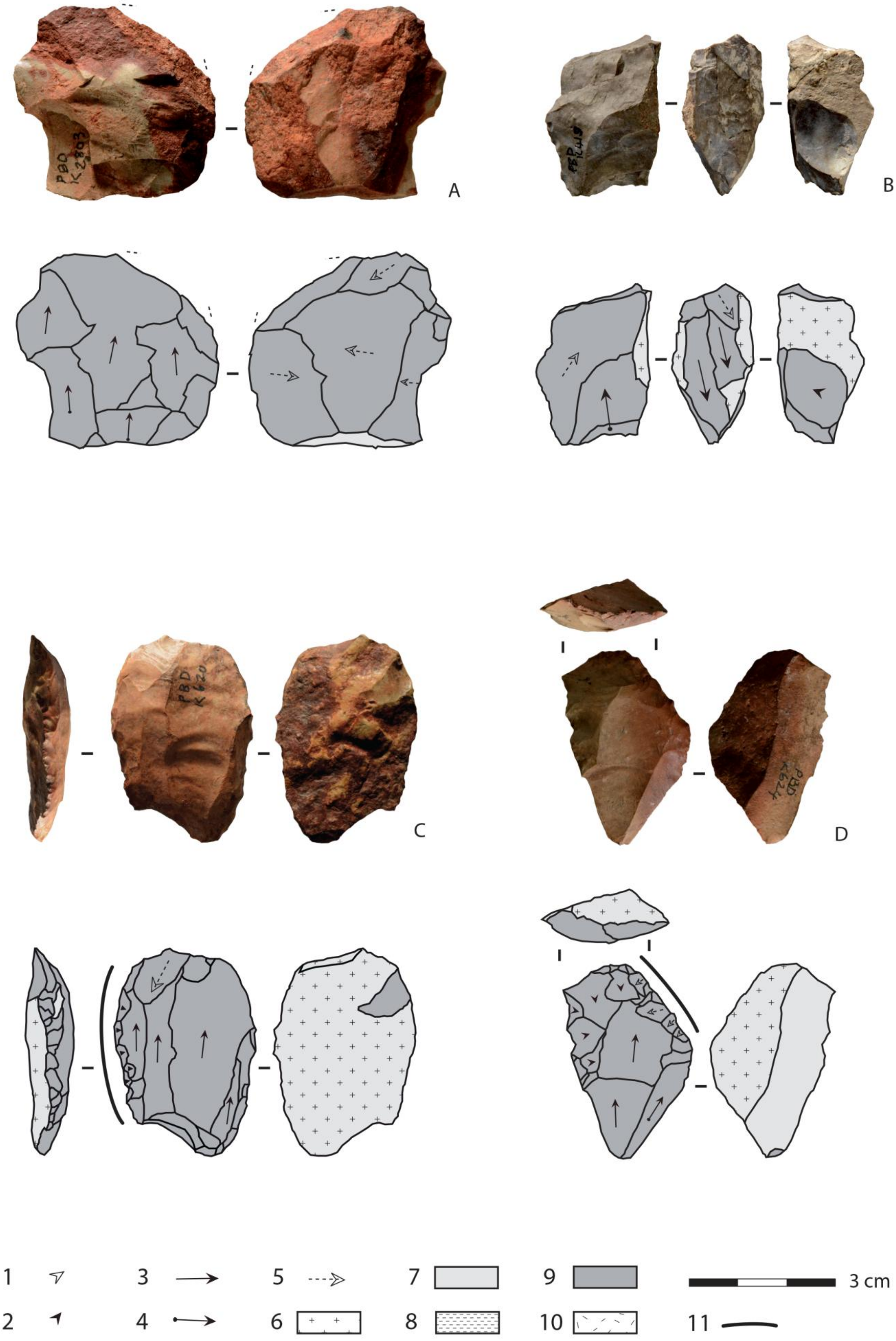

C

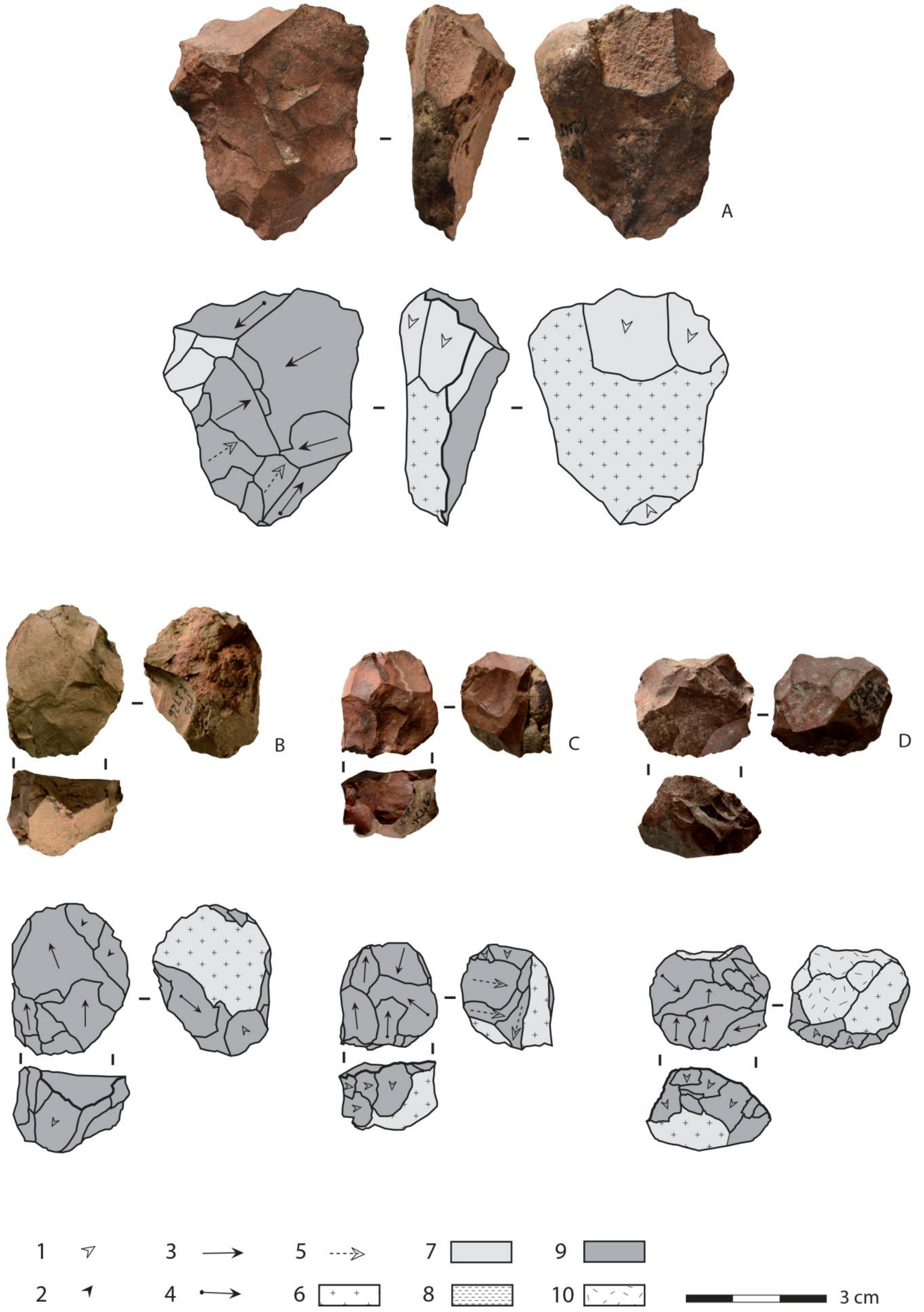

D

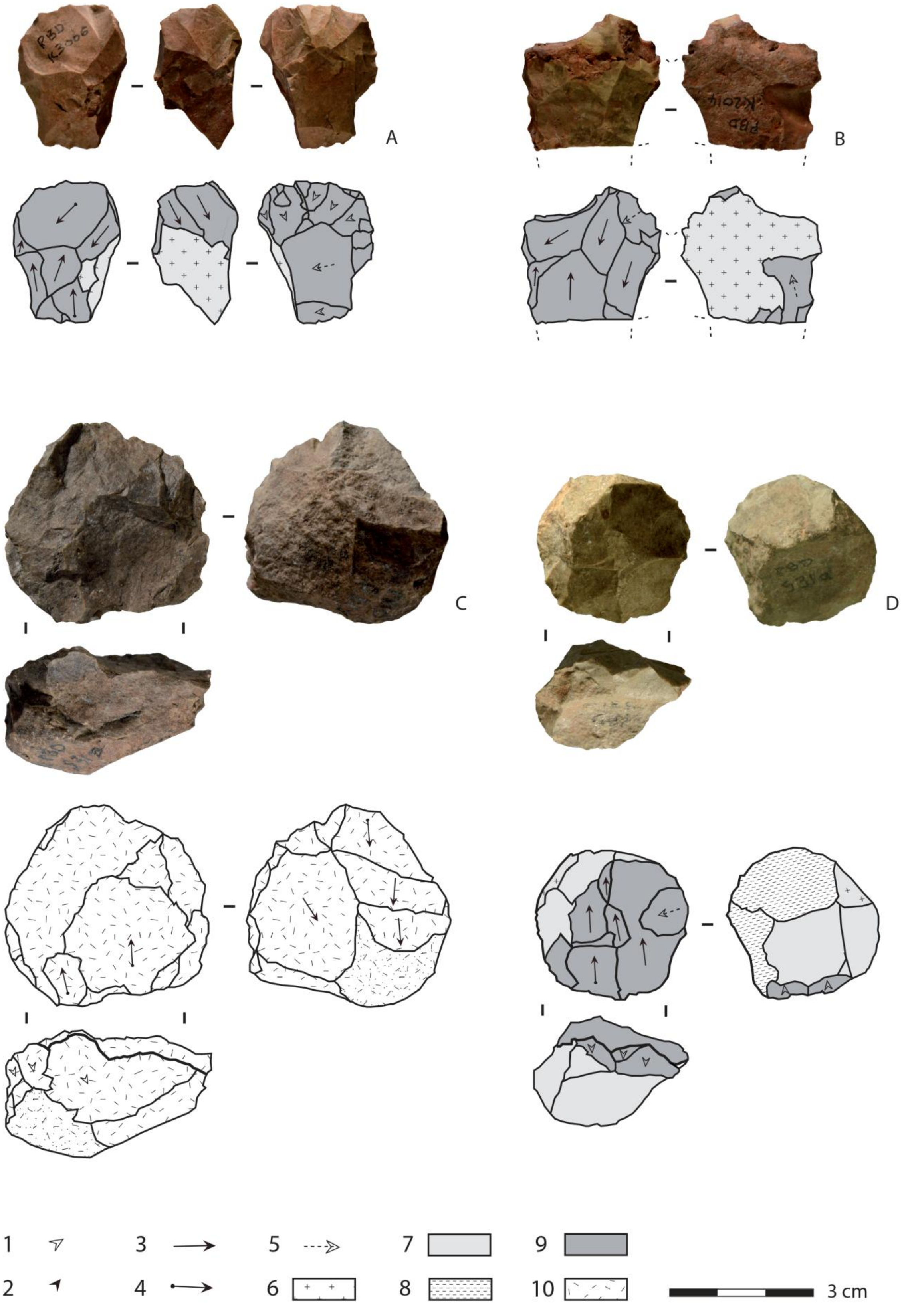

Supplement: S2 Fig — A, B, C, D: Picture and technological drawing of each core. Caption for drawings: 1. knapping platform preparation, 2. convexity preparation, 3. blade removal without initiation, 4. blade removal with initiation, 5. indeterminate removal, 6. cortex or patina, 7. pre-heating surface, 8. heat-induced fracture, 9. post-heating removal, 10. burnt after discard, potlids, 11. retouch. (PDF) [file pone.0163874.s002.pdf]
